# MRI Insights Into Adolescent Neurocircuitry—A Vision for the Future

**DOI:** 10.3389/fnhum.2020.00237

**Published:** 2020-07-07

**Authors:** Olga Tymofiyeva, Vivian X. Zhou, Chuan-Mei Lee, Duan Xu, Christopher P. Hess, Tony T. Yang

**Affiliations:** ^1^Department of Radiology and Biomedical Imaging, University of California, San Francisco, San Francisco, CA, United States; ^2^Division of Child and Adolescent Psychiatry, Department of Psychiatry and Behavioral Sciences, Weill Institute for Neurosciences, University of California, San Francisco, San Francisco, CA, United States; ^3^Clinical Excellence Research Center, Stanford University, Stanford, CA, United States

**Keywords:** MRI, adolescence, brain connectivity, psychiatric disorders, depression

## Abstract

Adolescence is the time of onset of many psychiatric disorders. Half of pediatric patients present with comorbid psychiatric disorders that complicate both their medical and psychiatric care. Currently, diagnosis and treatment decisions are based on symptoms. The field urgently needs brain-based diagnosis and personalized care. Neuroimaging can shed light on how aberrations in brain circuits might underlie psychiatric disorders and their development in adolescents. In this perspective article, we summarize recent MRI literature that provides insights into development of psychiatric disorders in adolescents. We specifically focus on studies of brain structural and functional connectivity. Ninety-six included studies demonstrate the potential of MRI to assess psychiatrically relevant constructs, diagnose psychiatric disorders, predict their development or predict response to treatment. Limitations of the included studies are discussed, and recommendations for future research are offered. We also present a vision for the role that neuroimaging may play in pediatrics and primary care in the future: a routine neuropsychological and neuropsychiatric imaging (NPPI) protocol for adolescent patients, which would include a 30-min brain scan, a quality control and safety read of the scan, followed by computer-based calculation of the structural and functional brain network metrics that can be compared to the normative data by the pediatrician. We also perform a cost-benefit analysis to support this vision and provide a roadmap of the steps required for this vision to be implemented.

## Introduction

Adolescence is the time of onset of many psychiatric disorders. Half of pediatric patients present with comorbid psychiatric disorders that complicate both their medical and psychiatric care. Currently, diagnosis and treatment decisions are based on symptoms. The field urgently needs brain-based diagnosis and personalized care.

Neuroimaging can shed light on how aberrations in brain circuits might underlie psychiatric disorders and their development in adolescents. MRI has become the leading modality for mapping the human brain non-invasively. Apart from mapping individual brain regions, the importance of connections between these regions and their role within the *brain network as a whole* are becoming increasingly recognized and studied within the framework of *connectomics* (Sporns et al., 2005). Different MRI techniques can be applied to map the connections of the network and to quantify the connectivity strength. Most commonly, connections are derived from diffusion-weighted images (with tractography used to model white matter pathways) or from the functional MRI (fMRI) signal (using temporal correlation of the signal as a proxy for connectivity). Connections can be compared between subjects individually. However, one can also utilize *graph theory*, which offers new ways to perform network characterization and comparison. Graph theory operates with an abstracted notion of a graph, which is defined as a set of nodes (in our case, brain regions), connected by a set of edges (e.g., white matter tracts). Important network characteristics can be extracted, such as node degree, characteristic path length, average clustering coefficient and other quantifiable measures of network connectivity (Rubinov and Sporns, 2010). Studying the human connectome using graph theory offers a unique opportunity to better understand inter-individual differences in the neural circuitry.

MRI connectomics has been applied to both the adult and developing brain (Hagmann et al., 2010), including extensive work by our group (Tymofiyeva et al., 2012, 2013, 2014; Ziv et al., 2013). This framework also has been applied to study the neural signature of psychiatric disorders, for example, adult depression (Bai et al., 2012; Korgaonkar et al., 2014; Qin et al., 2014; Gong and He, 2015; Sacchet et al., 2016), as well as adolescent depression (Ellis et al., 2017; Tymofiyeva et al., 2017) and anxiety (Sharp and Telzer, 2017). In the next section, we systematically review both the structural and functional connectivity literature on psychiatric disorders and related symptoms in adolescents.

## Review of Connectivity Studies in Adolescents

To perform a review of MRI literature over the last 5-years that provides insights into development of psychiatric disorders in adolescents, the electronic database PubMed was searched using the following Boolean search term, applied to titles and abstracts:

*(MRI OR fMRI OR DTI) AND (adolescent OR youth) AND (psychiatric OR neurologic OR mental OR depression OR autism OR anxiety OR PTSD OR psychosis OR ADHD OR attention OR bipolar OR schizophrenia OR OCD) AND (connectivity OR connectome OR network OR circuit)*.

We thus focused on eight disorders: major depressive disorder (MDD), autism, anxiety, bipolar disorder, attention-deficit/hyperactivity disorder (ADHD), post-traumatic stress disorder (PTSD), schizophrenia, and obsessive-compulsive disorder (OCD) or their relevant clinical and non-clinical symptoms in youth. We defined the age of adolescence as age between 10 and 19, whereas studies could also include older subjects in addition to those between 10 and 19.

The initial search resulted in 177 entries. After excluding articles that: (a) are not in English (1 article), (b) animal studies (5); (c) review articles (8), (d) focus on non-general populations such as Down syndrome (37), (e) do not include any pathology focus (11), (f) do not analyze brain connectivity (21), the resulting set comprised 94 articles. Two additional articles meeting the eligibility criteria were identified through other sources. The total number of articles included in qualitative synthesis was 96. The search results and main finding of the included articles are summarized in Table 1.

**Table 1:** Summary of the brain connectivity studies that provide insights into development of psychiatric disorders in adolescents published the last 5-years.

**References**	**# of subjects**	**Subject age in Years (±std. dev)**	**Disorder/Trait**	**MRI method(s)**	**Diagnosis/Future risk/Predicting treatment response/Treatment effects**	**Group difference vs. Single subject**	**Main MRI findings**
Adluru et al. (2017)	100	13–18	Anxiety	DTI	Diagnosis	Group difference	Within monozygotic twin pairs, the more anxious twin exhibited decreased significantly decreased FA and axial diffusivity in the left uncinate fasciculus, compared to the less anxious twin
Alarcón et al. (2018)	40	15–18	Non-clinical: rumination (relating to depression)	Task-based fMRI (conflict between self-referential processing and cognitive control)	Future risk	Group difference	Girls displayed stronger fc of frontoparietal network (FPN) and DMN during self-referential processing (SRP) relative to boys. Co-rumination, which was the only self-reported measure that differentiated boys and girls, mediated cognitive control performance during SRP Incongruent conditions
Balevich et al. (2015)	99	15.9 ± 1.7 (adol-S) 17.1 ± 2.1 (adol-C) 43.7 ± 10.2 (adult-S) 42.2 ± 11.5 (adult-C)	Schizophrenia	DTI	Diagnosis	Group difference	Compared to healthy controls, both adult and adolescent patients with schizophrenia showed reduced anisotropy of the corpus callosum; however, adolescent patients showed reductions primarily in anterior regions, whereas adult patients showed more prominent reductions in posterior regions
Bebko et al. (2015)	60	8–17	Bipolar disorder ADHD Anxiety	Resting state fMRI	Diagnosis	Group difference	This study examined relationships among symptom dimensions, diagnostic categories, and rsFC in behaviorally and emotionally dysregulated youth. Two dimensional measures showed significant inverse relationships with rsFC regardless of diagnosis: 1) PGBI-10M (mania, depression, and anxiety severity) with amygdala-left posterior insula/bilateral putamen, and 2) depressive symptoms with amygdala-right posterior insula connectivity. Diagnostic categories showed no significant relationships with rsFC. rsFC between the amygdala and posterior insula decreased with increasing severity of behavioral and emotional dysregulation and depression
Bédard et al. (2014)	45	9–15	ADHD	Task-based fMRI (N-back test of working memory for spatial position)	Diagnosis	Group difference	Compared to healthy controls, youth with ADHD showed greater functional connectivity between the left dorsolateral PFC and left intraparietal sulcus and reduced left dorsolateral PFC connectivity with left mid-cingulate cortex and PCC for the high load contrast. Reanalysis with a more conservative statistical approach showed group differences in dorsolateral PFC-mid-cingulate connectivity
Boets et al. (2018)	34	11–18	Autism	DTI Global probabilistic tractography	Diagnosis	Group difference	Compared to the control group, the ASD group showed reduced FA in the right and left inferior longitudinal fasciculus (ILF) and increased radial diffusivity in the right ILF Lower FA in the right ILF showed a slight association with the presence of more self-reported ASD characteristics
Chang et al. (2017)	79	9–18	Non-clinical: high risk for bipolar disorder	Task-based fMRI (implicit emotion perception task)	Future risk	Group difference	High-risk youth showed greater fc between the right amygdala and ventrolateral PFC, and the visual cortical regions, compared to healthy controls
Chattopadhyay et al. (2017)	116 (cross-sectional) 47 (longitudinal)	11–17	Depression	Resting state fMRI	Diagnosis Treatment effects	Group difference	Compared to controls, depressed adolescents showed significantly greater rsFC to the left amygdala and bilateral supragenual ACC, but no difference in connectivity to the PFC. Treatment effects were observed in the right insula connected to the left supragenual ACC, with baseline case-control differences reduced and no concomitant differences in areas of cognitive control neural systems. rsFC changes were significantly correlated with changes in depression severity
Chen et al. (2017)	109	15.6 ± 1.8 (S) 15.4 ± 1.6 (S-C) 13.1 ± 3.1 (A) 12.9 ± 2.9 (A-C)	Schizophrenia Autism	Resting state fMRI	Diagnosis	Single subject Group difference	Classification between individuals with disorders and healthy controls was achieved with high accuracy. Shared atypical brain connections contributing to classification were mostly present in the DMN and SN. These functional connections were significantly associated with severity of social deficits in ASD. Distinct atypical connections were also more related to the DMN and SN, but showed different atypical connectivity patterns between the two disorders
Chuang et al. (2017)	140	11–18	Depression	Task-based fMRI (affective go/no-go task)	Diagnosis	Group difference	Compared to healthy male adolescents, depressed male adolescents showed decreased activation in the cerebellum with significant group-by-age interaction in connectivity
Cisler et al. (2018)	88	11–17	Non-clinical: early life trauma exposure (relating to PTSD)	Task-based fMRI (facial emotion processing task)	Future risk	Group difference	In healthy adolescent females, self-reported early life trauma was significantly associated with greater modularity, greater assortativity, and lesser global efficiency during facial emotion processing—even when controlling for PTSD symptom severity. Beyond the effect of early life trauma, PTSD diagnoses were associated with greater modularity. Individual differences in large-scale network modularity were predictive of both the degree of bilateral amygdala functional activation during the task, as well as degree of functional connectivity between the amygdala and medial PFC
Cisler et al. (2016)	20	11–16	PTSD	Task-based fMRI (facial emotion processing task)	Predicting treatment response Treatment effects	Group difference	Pre-treatment (trauma-focused CBT) individual differences in modularity, assortativity, and global efficiency during covert fear vs. neutral task blocks predicted PTSD symptom reduction. PTSD patients with greater treatment response showed greater network modularity and assortativity but lesser efficiency. At the group level, greater symptom reduction was associated with greater pre-to-post-treatment increases in network assortativity and modularity, and this was more pronounced among participants with less symptom improvement
Cisler et al. (2013)	30	12–16	PTSD Non-clinical: assaultive violence exposure	Task-based fMRI (presentation of fearful vs. neutral facial expression images)	Diagnosis Future risk	Group difference	Within the frontocingulate network, PTSD severity was associated with weakened functional connectivity between the left amygdala and the perigenual anterior cingulate. Within the frontoparietal network, assaulted girls demonstrated weakened connectivity of the premotor cortex with the right middle frontal gyrus. Within the DMN, assault exposure and PTSD severity were associated with strengthened functional connectivity of the parahippocampus with the medial and lateral prefrontal cortex, respectively. Individual differences in functional connections within the frontocingulate network and frontoparietal network among the assaulted group were strongly associated with caregiver-rated family disengagement
Clasen et al. (2014)	24	13–15	Non-clinical: familial risk for depression	Resting state fMRI	Future risk	Group difference	High-risk adolescents with a parental history of depression showed lower levels of functional connectivity between a right inferior prefrontal region and other critical nodes of the attention control network, including the right middle frontal gyrus and right supramarginal gyrus. Among high-risk adolescents, increased severity of parents' worst episode of depression was associated with altered cognitive control network connectivity in adolescents
Cullen et al. (2016)	13	12–19	Depression	Resting state fMRI	Treatment effects	Group difference	Analysis of change in amygdala rsFC showed that treatment response was associated with increased amygdala rsFC with right frontal cortex, but decreased amygdala rsFC with right precuneus and right PCC
Das et al. (2013)	58	15.1 ± 0.34	Non-clinical: sub-clinical emotional syndromes	Task-based fMRI (image-based emotion processing task)	Future risk	Group difference	Between groups, the hippocampus showed a pattern of reverse coupling with the amygdala and insula that significantly correlated with trait anxiety
Davey et al. (2015)	56	16.5 ± 0.5 (baseline) 18.8 ± 0.5 (follow-up)	Depression	Resting state fMRI	Future risk	Group difference	Adolescents with no history of mental illness received an fMRI scan at baseline and a follow-up scan 2-years later. Magnitude of amygdala connectivity with sgACC showed significant positive correlation with negative affectivity at both time points. Change in amygdala-sgACC connectivity between assessments was correlated with change in negative affectivity. Of the 56 participants in the study, eight developed a first episode of depression between the baseline and follow-up assessments; they showed increased amygdala-subgenual connectivity at follow-up
Diwadkar et al. (2014)	46	14.1 ± 3.1 (R) 15.4 ± 2.7 (C)	Non-clinical: familial risk for schizophrenia or bipolar disorder	Task-based fMRI (attention and visual control tasks)	Future risk	Group difference	Compared to healthy controls, higher-risk adolescents were characterized by significant reductions in coupling across both frontal-striatal and frontal-parietal pathways
Dorfman et al. (2016)	71	13.2 ± 2.7 (A) 13.0 ± 2.7 (C)	Anxiety	Resting state fMRI	Diagnosis	Group difference	Compared to healthy controls, anxious adolescents showed abnormally low intrinsic functional connectivity within the striatum (between the nucleus accumbens and caudate nucleus) and between the striatum and prefrontal regions (including the sgACC, posterior insula, and supplementary motor area)
Ellis et al. (2017)	243	17–19	Non-clinical: depressive symptoms	DTI and tractography	Future risk	Group difference	Adolescents that experienced increasing depression symptoms in early adolescence showed differences in several frontal and temporal brain regions, compared to adolescents with stable low levels of depression. Affected tracts corresponded to areas of white matter that are still maturing during adolescent, particularly frontolimbic regions
Fitzgerald et al. (2014)	63	8–19	OCD	DTI	Diagnosis	Group difference	Compared to healthy controls, patients with OCD showed more pronounced age-related increases in FA in the anterior corpus callosum, anterior cingulum bundle, and anterior limb of the internal capsule, as well as several other white matter tracts. Among OCD patients, greater FA in the anterior cingulum bundle correlated with more severe symptoms after controlling for age
Fowler et al. (2017)	41	15.42 ± 0.33	Non-clinical: stress-reactive rumination (relating to depression)	Task-based fMRI (emotion regulation task and social stress task)	Future risk	Group difference	Positive functional connectivity between the amygdala and ventrolateral PFC during the emotion regulation task mediated the association between stress-reactive rumination and depressive symptoms
Friedman et al. (2017)	54	9–21 (O) 12–21 (C)	OCD	Task-based fMRI (uni-manual motor task)	Diagnosis	Group difference	OCD subjects were characterized by hyper-modulation by the dorsal ACC. Dynamically-driven task demands during simple uni-manual motor control induced compensatory network interactions in cortical-thalamic regions in OCD
Fryer et al. (2019)	125	11–29	Schizophrenia Psychosis	Task-based fMRI (go/no-go task)	Diagnosis Future risk	Group difference	Compared to the healthy control group, the early schizophrenia and clinical high risk (CHR) groups showed significantly less coupling during NoGo trials relative to Go trials between the ACC and the bilateral medial PFC, PCC, and precuneus
Gao et al. (2014)	35	10–18	Bipolar disorder	Resting state fMRI	Diagnosis	Group difference	Compared to healthy controls, depressed adolescents with bipolar disorder showed decreased regional homogeneity in the medial frontal gyrus, bilateral middle frontal gyrus and middle temporal gyrus, and right putamen. Depressed adolescents with bipolar disorder had significant negative correlations of mood and feelings questionnaire scores with mean regional homogeneity values in the medial frontal gyrus and right middle frontal gyrus
Geng et al. (2016)	57	13–17	Depression	Resting state fMRI DTI	Diagnosis	Group difference	Compared to healthy controls, adolescents with depression showed significantly lower FA values in the fornix and decreased functional connectivity in four PFC regions. Among healthy controls, significant negative correlations were observed between fornix FA values and hippocampus-PFC functional connectivity. Among adolescents with depression, no significant correlation was found between the fornix FA and the strength of functional connectivity
Gold et al. (2016)	82	Youth: 14.76 ± 2.82 (A) 14.42 ± 2.62 (C) Adults: 32.90 ± 6.97 (A) 29.1 ± 7.50 (C)	Anxiety	Task-based fMRI (extinction recall task)	Diagnosis	Group difference	Whole-brain analyses showed significant interactions of anxiety, age, and attention task (threat appraisal, explicit threat memory, physical discrimination) on left amygdala functional connectivity with the ventral medial PFC and ventral ACC. During threat appraisal and explicit threat memory, anxious youth showed more negative amygdala-PFC coupling, whereas anxious adults showed more positive coupling
Green et al. (2017)	38	9–17	Autism	Exposure-based fMRI (mildly aversive auditory and tactile stimuli)	Diagnosis	Group difference	Compared to healthy controls, ASD subjects showed aberrant modulation of fc between pulvinar and cortex (including sensory-motor and prefrontal regions) during sensory stimulation. Among ASD subjects, pulvinar-amygdala connectivity was correlated with severity of sensory over-responsivity symptoms
Green et al. (2016)	61	8–17	Autism	Resting state fMRI Exposure-based fMRI (mildly aversive tactile and auditory stimuli)	Diagnosis	Group difference	Sensory over-responsivity in youth with ASD was related to increased rsFC between SN nodes and brain regions implicated in primary sensory processing and attention. The strength of this connectivity at rest was related to the extent of brain activity in response to auditory and tactile stimuli
Gruner et al. (2014)	46	9–17	OCD	Resting state fMRI	Diagnosis	Single subject	Independent component analysis identified three components that maximally separated healthy controls from OCD adolescents: a middle frontal/dorsal anterior cingulate network, an anterior/posterior cingulate network, and a visual network—yielding an overall group classification of 76.1%. Compared to healthy controls, OCD patients showed significantly higher independent component expression scores in the middle frontal/dorsal anterior cingulate and anterior/posterior cingulate networks, but lower within the visual network. Among OCD patients, higher scores in the anterior/posterior cingulate network correlated with greater severity of compulsions among patients
Guo et al. (2016)	65	12–18	Autism	Resting state fMRI	Diagnosis	Group difference	Compared to healthy controls, adolescents with ASD showed decreased fc between the amygdala and subcortical regions, including the bilateral thalamus and right putamen
Hafeman et al. (2017)	78	8–16	Bipolar disorder ADHD	Task-based fMRI (implicit emotion processing task)	Diagnosis	Group difference	Fc between amygdala and left ventrolateral PFC in response to emotions vs. shapes significantly differed by group. Bipolar subjects showed positive functional connectivity (emotions > shapes), healthy controls showed inverse functional connectivity (emotions < shapes), and ADHD subjects showed intermediate functional connectivity. A significant group × emotion interaction was found in amygdala-subgenual cingulate fc, explained by differences in fc in response to negative emotions. Amygdala-subgenual fc was also positively associated with depressive symptoms and stimulant medication
Hamm et al. (2014)	56	13.9 ± 3.1 (A) 14.6 ± 3.9 (C)	Anxiety	Resting state fMRI	Diagnosis	Group difference	Compared to healthy controls, youth with anxiety disorders showed hyperconnectivity between the right amygdala and insula, and hypoconnectivity between the left amygdala and the ventromedial PFC and PCC. Among youth with anxiety disorders, connectivity was not correlated with anxiety severity
Harlalka et al. (2018)		9–12 (children) 13–16 (adolescents)	Autism	Resting state fMRI	Diagnosis	Group difference	Compared to healthy controls, children and adolescents with ASD showed increased functional integration at the expense of decreased functional segregation. Adolescents with ASD showed significant decrease in modularity and increase in participation coefficient, and significant hypoconnectivity in the DMN. In contrast, children with ASD showed both hyper- and hypoconnectivity
Henje Blom et al. (2015)	67	13–18	Depression	Task-based fMRI (facial emotion processing task)	Diagnosis	Group difference	Compared to healthy controls, adolescents with depression showed greater fc between the anterior/middle insular cortex and the right fusiform gyrus, left middle frontal gyrus, and right amygdala/parahippocampal gyrus
Ho et al. (2017)	95	13–18	Depression	Resting state fMRI Task-based fMRI (response inhibition task)	Diagnosis	Group difference	Compared to controls, adolescents with depression showed inflexibility in local efficiency of the right dorsal ACC/medial frontal gyrus (MFG). Individual differences in flexibility (limited task-evoked vs. resting-state connectivity) of local right dorsal ACC/MFG significantly predicted inhibition performance, and reduced local efficiency of the dorsal ACC/MFG during a response inhibition task was significantly associated with an earlier age of depression onset
Ho et al. (2014)	38	15.8 ± 1.4 (D) 16.1 ± 1.2 (C)	Depression	Task-based fMRI (implicit fear facial affect recognition task)	Diagnosis	Group difference	Compared to healthy controls, adolescents with depression showed significantly increased sgACC-amygdala functional connectivity and decreased sgACC-fusiform gyrus, sgACC-precuneus, sgACC-insula, and sgACC-middle frontal gyrus functional connectivity. Among depressed adolescents, sgACC-precuneus fc was significantly negatively correlated with depression severity. Compared to healthy controls, depressed adolescents showed poorer perceptual sensitivity in the task, and individual differences in perceptual sensitivity significantly correlated with sgACC fc and depression scores
Hong et al. (2017)	184	Children: 8.60 ± 0.75 (ADHD) 8.68 ± 0.65 (C) Teens: 13.17 ± 1.94 (ADHD) 12.21 ± 2.12 (C) Adults: 26.77 ± 2.54 (ADHD) 24.94 ± 1.41 (C)	ADHD	Resting state fMRI	Diagnosis	Group difference	The degree centrality of the left middle temporal gyrus showed significant interaction effects between disease status (ADHD or healthy control) and age (child, adolescent, or adult). Other regions with significant interaction effects included the left superior frontal gyrus, left inferior frontal gyrus, right inferior frontal gyrus, right precentral gyrus, left superior temporal gyrus, left middle temporal gyrus, right postcentral gyrus, left insular gyrus, left medioventral occipital cortex, right medioventral occipital cortex, left amygdala, and left basal ganglia
Hulvershorn et al. (2014)	63	6–13	ADHD	Resting state fMRI	Diagnosis	Group difference	Among youth with ADHD, higher emotional lability ratings were associated with greater positive intrinsic fc between the amygdala and rostral ACC, and negatively associated with intrinsic fc between bilateral amygdala and posterior insula/superior temporal gyrus. Patterns of amygdala-cortical intrinsic fc in ADHD youth with low emotional lability did not differ from the comparison group, and the effect sizes for these comparisons were smaller than those for the trend-level differences observed between the high emotional lability group and the healthy control group
Hwang et al. (2015)	61	14.53 ± 2.00 (A) 13.91 ± 2.13 (C)	ADHD	Task-based fMRI (affective Stroop paradigm)	Diagnosis	Group difference	Compared to healthy controls, ADHD patients showed reduced fc between the dorsomedial frontal cortex and lateral frontal cortex during congruent and incongruent task trials relative to view trials. Among ADHD patients, decreased connectivity between the amygdala and lentiform nucleus was found in the presence of emotional stimuli
Iadipaolo et al. (2018)	55	6–17	Non-clinical: resilience (relating to depression)	Resting state fMRI	Future risk	Group difference	Children and adolescents with higher trait resilience spent a significantly lower fraction of total time in a dynamic rsFC state characterized by increased rsFC between the anterior DMN and right CEN. Within this state, resilience was significantly associated with reduced SN rsFC with the anterior DMN and right CEN. More resilient youth reported lower depressive symptoms, but the effects of resilience on rsFC were independent of depressive symptoms and adversity exposure
Jack and Morris (2014)	30	14.20 ± 1.61 (A) 13.80 ± 1.70 (C)	Autism	fMRI imitation paradigm	Diagnosis	Group difference	Among adolescents with ASD, stronger psychophysiological interactions between Crus I of neocerebellum and right posterior superior temporal sulcus were associated with greater mentalizing abilities
Jacobs et al. (2016)	22	15.41 ± 1.97 (RFCBT) 15.69 ± 1.89 (control)	Depression	Resting state fMRI	Treatment effects	Group difference	This study was a pilot randomized control trial investigating the effects of rumination-focused cognitive behavior therapy (RFCBT) on reducing rumination and residual depressive symptoms in adolescents with a history of depression and risk of relapse. Over the course of 8 weeks, adolescents who received RFCBT showed significantly reduced rumination and self-reported depression, as well as significant decreases in connectivity between the left PCC and right inferior frontal gyrus and bilateral inferior temporal gyri. Degree of change in connectivity was correlated with changes in self-reported depression and rumination
Jacobs et al. (2014)	53	18–23	Depression	Task-based fMRI (go/no-go task)	Diagnosis	Group difference	Compared to healthy controls, unmedicated adolescents with remitted depression showed hyperconnectivities from both PCC and sgACC seeds with lateral, parietal, and frontal regions of the CEN, extending to the dorsal medial wall. A factor analysis reduced extracted data and a PCC factor was inversely correlated with rumination among depressed adolescents. Two factors from the sgACC hyperconnectivity clusters were related to performance in cognitive control on a Go/No-Go task, one positively and one inversely
Jacobson McEwen et al. (2014)	25	11–13	Psychosis	Resting state fMRI	Diagnosis	Group difference	Compared to healthy controls, adolescents with psychotic symptoms showed reduced intrinsic fc between the right inferior frontal gyrus and the cingulate, between the right inferior frontal gyrus and the striatum, between the anterior cingulate and claustrum, and between the precuneus and supramarginal gyrus. Compared to healthy controls, adolescents with psychotic symptoms showed stronger intrinsic fc between the superior frontal gyrus and claustrum, and between the inferior frontal gyrus and the lingual gyrus
James et al. (2016)	61	13–18	Schizophrenia	DTI and tractography	Diagnosis	Group difference	Compared to healthy controls, patients with adolescent-onset schizophrenia showed generalized cognitive impairment with specific deficits in verbal learning and memory, and in processing speed. These measures correlated positively with dorsolateral PFC connectivity with the striatum, and with IQ. DTI voxel-wise comparisons showed lower connectivity between striatum and the motor and lateral orbitofrontal cortices bilaterally, the left amygdala-hippocampal complex, right ACC, left medial orbitofrontal cortex, and right dorsolateral PFC in adolescent-onset schizophrenia patients relative to healthy controls
Jann et al. (2015)	39	13.8 ± 2.0 (A) 12.8 ± 3.6 (C)	Autism	Resting state fMRI	Diagnosis	Group difference	Increased local fc in the anterior module of the DMN was accompanied by decreased cerebral blood flow in the same area. Both alterations were associated with greater social impairments. While fc was correlated with cerebral blood flow in healthy controls, this association was disrupted in ASD youth. Reduced long-range fc between the anterior and posterior modules of the DMN was also found in ASD youth
Jarcho et al. (2015)	90	8–17 (adolescents) 18–49 (adults)	Non-clinical: social anxiety	Task-based fMRI (prediction and social feedback task)	Future risk	Group difference	For socially anxious adolescents, but not anxious adults or healthy controls of either age groups, social evaluation prediction errors elicited heightened negative fronto-striatal fc
Jin et al. (2017)	229	15 ± 0.583	Non-clinical: sensitivity to loss (relating to depression)	Task-based fMRI (monetary gambling task with win and loss feedback)	Future risk	Group difference	Increased OFC-posterior insula connectivity during loss was marginally associated with higher concurrent depressive symptoms and significantly associated future depressive symptoms, but this relationship was not moderated by parental history of depression. In contrast, OFC connectivity changes in response to win did not predict concurrent or future depression symptoms
Johnston et al. (2017)	68	14–25	Bipolar disorder (suicide attempts)	DTI Task-based fMRI (emotion processing task)	Future risk	Group difference	Compared to bipolar adolescents and young adults without a history of suicide attempts, patients with prior suicide attempts showed significant reductions in: white matter integrity in the uncinate fasciculus, ventral frontal, and right cerebellum regions; and amygdala fc to the left ventral and right rostral PFC. Among attempters, significant negative associations were found between right rostral prefrontal connectivity and suicidal ideation, and between left ventral prefrontal connectivity and attempt lethality
Joshi et al. (2017)	31	15–29	Autism	Resting state fMRI	Diagnosis	Group difference	Compared to healthy controls, the ASD group showed a weaker pattern of positive intra-DMN and negative extra-DMN rsFC correlations. The strength of intra-DMN coupling was significantly reduced with the medial PFC and the bilateral angular gyrus regions, and the polarity of the extra-DMN correlation with the right hemispheric task-positive regions of fusiform gyrus and supramarginal gyrus was reversed from typically negative to positive
Kaczkurkin et al. (2018)	120	11–23	Psychosis (overall psychopathology)	Resting state fMRI	Future risk	Group difference	Overall psychopathology was associated with decreased fc between the dorsal ACC and bilateral caudate
Keding and Herringa (2016)	53	8–18	PTSD	Task-based fMRI (facial emotion processing task)	Diagnosis	Group difference	Connectivity analyses revealed paradoxical coupling in prefrontal–amygdala circuits, including dACC–dorsomedial (dm)PFC, amygdala–dmPFC, and amygdala–ventrolateral (vl)PFC. PTSD youth showed reduced connectivity in response to angry faces, but increased connectivity in response to happy faces, the reverse of healthy youth
Kim et al. (2016)	42	13–18	Depression	Resting state fMRI	Diagnosis	Group difference	Compared to healthy controls, depressed adolescents with disruptive behaviors showed lower rsFC from the amygdala to the orbitofrontal cortex and parahippocampal gyrus, as well as higher PCC rsFC in a cluster that included the left precentral gyrus, left insula, and left parietal lobe. Among depressed adolescents with disruptive behaviors, depression scores were negatively correlated with rsFC from the amygdala to the right orbitofrontal cortex, while disruptive behavior scores were positively correlated with rsFC from the PCC to the left insular cortex
Klimes-Dougan et al. (2018)	11	12–19	Depression	Resting state fMRI	Predicting treatment response	Group difference	This study assessed improvement in depression symptoms after 8 weeks of SSRI treatment. Higher levels of pre-treatment amygdala rsFC with the right central parietal opercular cortex and Heschl's gyrus predicted better treatment response. Higher levels of pre-treatment amygdala rsFC with the right precentral gyrus and with left SMA predicted a worse treatment response
Kujawa et al. (2016)	118	7–25	Anxiety	Task-based fMRI (facial emotion processing task)	Diagnosis	Group difference	Anxiety interacted with age to predict amygdala-ACC connectivity across emotional faces. Age was negatively correlated with connectivity among healthy controls, but was positively correlated among anxious subjects. Group effects were observed on amygdala connectivity with mid-cingulate and middle frontal gyri. Effects of anxiety and age on amygdala activation were not significant
LeWinn et al. (2018)	75	13–17	Depression	Task-based fMRI (cognitive reappraisal task)	Diagnosis	Group difference	Among adolescents with depression, reduced connectivity was found between the left dorsomedial PFC and the anterior insula/inferior frontal gyri bilaterally, and between the left dorsolateral PFC and left anterior insula/inferior frontal gyri
LeWinn et al. (2014)	94	13–17	Depression	DTI	Diagnosis	Group difference	Compared to healthy controls, adolescents with depression showed significantly lower FA and higher radial diffusivity in the bilateral uncinate fasciculus. No significant differences were observed in the cingulum. Tract-based spatial statistics showed lower FA values in the white matter associated with the limbic-cortical-striatal-thalamic circuit, corpus callosum, and anterior and superior corona radiata
Li et al. (2019)	65	12–18	Schizophrenia	Functional MRI	Diagnosis	Group difference	Compared to healthy controls, adolescent-onset schizophrenia (AOS) patients showed significantly decreased global efficiency of the brain functional network and reduced nodal efficiency and strength in the bilateral posterior parahippocampus, bilateral precuneus, and left hippocampus. In the left hippocampus of healthy controls, there were significant negative associations between nodal efficiency and age as well as between nodal strength and age, both of which were reversed in AOS patients. Reduced efficiency identified in the right posterior parahippocampus showed a negative correlation with illness duration within AOS patients
Manelis et al. (2015)	81	7–17	Non-clinical: familial risk for bipolar disorder	Task-based fMRI (facial emotion processing task)	Future risk	Group difference	Offspring of bipolar parents showed significantly more negative right amygdala-ACC fc in response to emotional faces vs. shapes, but significantly more positive right amygdala-left ventrolateral PFC fc in response to happy faces, in comparison to healthy controls and to offspring of parents with other psychopathology
Marusak et al. (2018)	42	6–17	Non-clinical: mindfulness (relating to anxiety)	Resting state fMRI	Future risk	Group difference	Trait mindfulness in adolescents relates to dynamic but not static rsFC. More mindful youth transitioned more between brain states over the course of the scan, spent overall less time in a certain connectivity state, and showed a state-specific reduction in connectivity between the SN and CEN. The number of state transitions mediated the link between higher mindfulness and lower anxiety
Marusak et al. (2017)	86	7–17	Non-clinical: early exposure to violence and/or abuse	Resting state fMRI	Future risk	Group difference	Trauma-exposed youth showed lower functional connectivity between the ventral tegmental area (VTA) and the hippocampus, compared to unexposed youth. No group differences in substantia nigra connectivity were observed. Increased anxiety symptoms were associated with reduced substantia nigra-nucleus accumbens connectivity
Morgan et al. (2016)	166	20	Depression	Task-based fMRI (monetary reward paradigm)	Diagnosis	Group difference	Compared to boys with no psychiatric history, boys with a history of depression showed heightened positive connectivity between the nucleus accumbens and the medial PFC when winning rewards relative to losing rewards. This altered fronto-striatal connectivity pattern was associated with a greater number of lifetime depressive episodes
O'Halloran et al. (2018)	818	14.55 ± 0.45 (normative) 14 ± 0.38 (ADHD) 14 ± 0.41 (C)	ADHD	Task-based fMRI (Stop Signal Task (SST))	Diagnosis	Group difference	In the normative dataset, good sustained attention was characterized by stronger negative fc between cerebellum and motor networks, while stronger positive fc within the motor network was a signature of poorer sustained attention. In separate samples, relative to controls, adolescents with ADHD symptoms had significantly higher intra-individual response variability (IRV) and stronger positive connectivity within low sustained attention networks associated with high IRV, as well as stronger positive connectivity within good sustained attention networks associated with low IRV. There were no differences between the groups for anti-correlated connections in networks associated with either high or low IRV
Ordaz et al. (2018)	40	14–17	Depression (suicidal ideation)	Resting state fMRI	Future risk	Group difference	Coherence of the lateral CEN, anterior DMN, and SN were significantly associated with lifetime severity of suicidal ideation. Only lateral CEN coherence was a significant predictor of lifetime suicidal ideation severity, was associated with current suicidal ideation, and was associated with a previously initiated suicide attempt
Osuch et al. (2014)	28	16–24	Non-clinical: repetitive non-suicidal self-injury	Exposure-based fMRI (painfully cold and comparison cool stimuli)	Future risk	Group difference	Reduced fc between the right orbitofrontal cortex and ACC was found in non-suicidal self-injury (NSSI) adolescents
Pan et al. (2017)	637	6–12	Depression	Resting state fMRI	Future risk	Group difference	Increased left ventral striatum node strength predicted increased risk for future depressive disorder. Among 11 reward network nodes examined, only the left ventral striatum significantly predicted depression. Striatal node strength did not predict anxiety, ADHD, or substance use
Pannekoek et al. (2014)	52	15.4 ± 1.5 (D) 14.7 ± 1.5 (C)	Depression	Resting state fMRI	Diagnosis	Group difference	Compared to healthy controls, adolescents with depression showed increased rsFC of the left amygdala with right parietal cortical areas, and decreased right amygdala rsFC with left frontal cortical areas and with right occipito-parietal areas. In depressed adolescents, the bilateral dorsal ACC showed decreased rsFC with the right middle frontal gyrus, frontal pole, and inferior frontal gyrus. No abnormalities in DMN rsFC were found, and differences in rsFC did not correlate with clinical measures
Paquola et al. (2017)	64	14–26	Non-clinical: childhood abuse, adulthood stress	Resting state fMRI	Future risk	Group difference	Worse psychiatric symptoms were significantly associated with higher levels of lifetime stress. Subjects with mismatched childhood and recent stress levels had reduced ACC-ventrolateral PFC rsFC, and greater ACC-hippocampus rsFC, compared to subjects with matched childhood and recent stress levels
Park et al. (2016)	52	<10 (child) 10–19 (teen)	ADHD	Resting state fMRI	Diagnosis	Group difference	In comparing brain connectivity patterns between child and adolescent ADHD patients, the DMN and frontoparietal networks showed significant group-wise connectivity pattern differences between child and adolescent ADHD patients
Patriat et al. (2016)	59	14.6 ± 2.6 (P) 14.0 ± 2.3 (C)	PTSD	Resting state fMRI	Diagnosis	Group difference	Compared to healthy controls, PTSD youth showed increased connectivity within the DMN (including increased PCC-inferior parietal gyrus connectivity) and age-related increases in PCC-ventromedial PFC connectivity. PTSD youth also showed greater anti-correlation between the PCC and multiple nodes within SN and attention control networks of the task-positive network. Among PTSD youth, DMN and task-positive network connectivity strength were positively and negatively associated, respectively, with re-experiencing symptoms of PTSD
Pitskel et al. (2014)	31	9–17	Autism	Task-based fMRI (cognitive reappraisal and emotional responses to disgusting images)	Diagnosis	Group difference	Compared to ASD youth, controls showed increased fc between the amygdala and ventrolateral PFC, as well as decreased functional connectivity between the amygdala and OFC, when down-regulating disgust
Platt et al. (2015)	30	15–17	Depression	Task-based fMRI (reappraisal paradigm)	Diagnosis	Group difference	During fMRI, subjects attended to and implemented reappraisal techniques in response to rejection. Reappraisal reduced negative mood and belief in negative thoughts in both depressed adolescents and healthy controls; however, during reappraisal trials depressed adolescents showed greater connectivity between the right frontal pole and numerous subcortical and cortical regions
Price et al. (2016)	78	9–14	Anxiety	Task-based fMRI (dot-probe task)	Diagnosis	Group difference	Among clinically anxious adolescents completing a dot-probe ask to assess vigilance to threat, vigilance toward threat was positively associated with self-reported distraction and suppression. Fc between a right amygdala seed region and dorsomedial and right dorsolateral PFC regions was inversely related to self-reported suppression and distraction, and dorsolateral PFC-amygdalar connectivity mediated the relationship between attentional vigilance and real-world distraction
Price et al. (2014)	121	9–13	Anxiety	Task-based fMRI (dot-probe task)	Diagnosis	Group difference	Among adolescents with anxiety, reduced fc between the rostrodorsal ACC and left parahippocampus/hippocampus was associated with greater anxiety
Quinlan et al. (2017)	1288	13–15	Non-clinical: symptoms of hyperactivity, inattention, and/or conduct problems (relating to ADHD)	Task-based fMRI (viewing of dynamic angry and neutral facial expressions)	Diagnosis	Group difference	Amygdala-precuneus connectivity was associated with hyperactivity/inattention symptoms
Rosso et al. (2014)	36	10–19	OCD	DTI	Diagnosis	Group difference	Compared to healthy controls, patients with OCD had significantly lower FA in 7 white matter clusters, with over 80% of significant voxels in the bilateral frontal cortex and corpus callosum. No regions were found of significantly higher FA in patients relative to controls. OCD patients also had significantly higher radial diffusivity in the right frontal cortex and right body of the corpus callosum. Among patients, earlier age at onset of OCD correlated significantly with lower FA in the right thalamus and with higher radial diffusivity in the right corpus callosum. FA and radial diffusivity were not significantly associated with symptom severity
Rzepa and McCabe (2016)	35	13–18	Non-clinical: high risk for depression	Resting state fMRI	Future risk	Group difference	Compared to adolescents at low risk for depression, adolescents at high risk were found to have decreased rsFC between the amygdala and the pregenual ACC, hippocampus, and precuneus; between the pregenual ACC and the putamen; and between the dorsal medial PFC and the precuneus. High risk adolescents were also found to have increased rsFC between the pregenual ACC and the PFC, and between the amygdala and the temporal pole
Sacchet et al. (2016)	111	13–18	Depression	Resting state fMRI	Diagnosis	Group difference	Compared to healthy controls, depressed adolescents showed hypoconnectivity between large-scale brain networks. Depressed adolescents showed significantly reduced connectivity between a specific set of resting-state networks, including components of the attention network, CEN, SN, and DMN. Among depressed adolescents, longer duration of depression was significantly correlated with reduced connectivity in this set of network interactions, specifically with reduced connectivity between components of the dorsal attention network, and the dorsal attention network was also characterized by reduced intra-network connectivity
Sadeghi et al. (2017)	60	14–42 (A) 10–39 (C)	Autism	Resting state fMRI	Diagnosis	Single subject Group difference	In this study, screening for ASD was developed based on characteristics of functional networks. Local and global parameters of the brain functional network were first calculated using graph theory, and network parameters of ASD subjects were statistically compared to those of healthy controls. Significantly altered parameters were used as input features of the screening system, and performance of the system was verified using multiple classification techniques. The support vector machine showed an accuracy of 92%
Scheuer et al. (2017)	37	12–16	Non-clinical: escalating depression symptom expression	Resting state fMRI	Future risk	Group difference	Compared to controls, adolescents with ≥ 10 point increase in depression scale t-scores (as assessed by the Childhood Depression Inventory) over time (range: 6–54 months) had decreased rsFC between the right amygdala and left inferior frontal supramarginal gyrus and right mid-cingulate cortex, and increased rsFC between the left amygdala and cerebellum
Singh et al. (2014)	49	8–17	Non-clinical: high risk for bipolar disorder	Resting state fMRI	Future risk	Group difference	Compared to low-risk youth (no personal or family psychopathology), high-risk youth (offspring of a parent with bipolar disorder) showed increased connectivity in the ventrolateral PFC subregion of the left CEN, which includes frontoparietal regions critical for emotion regulation. Compared to low-risk youth, high-risk youth also showed decreased connectivities between the left amygdala and pregenual cingulate, between the subgenual cingulate and supplementary motor cortex, and between the left ventrolateral PFC and left caudate. High-risk youth showed stronger connections in the ventrolateral PFC with age and higher functioning, and weaker connections between the left ventrolateral PFC and caudate with more family chaos
Stoddard et al. (2016)	117	10–50	Bipolar disorder	Resting state fMRI	Diagnosis	Group difference	Compared to healthy controls, bipolar adolescents and adults showed areas of dysconnectivity across the brain, comprising two networks—temporal and parietal areas involved in late stages of visual processing, and corticostrial areas involved in attention, cognitive control, and response generation. No significant age-group by diagnosis interactions were found
Straub et al. (2017)	38	13–18	Depression	Resting state fMRI	Diagnosis Treatment effects Predicting treatment response	Group difference	In comparing healthy controls and adolescents with depression prior to group CBT, patients with depression showed stronger amygdala and sgACC connectivity with regions of the DMN, whereas healthy controls showed stronger seed-based connectivity with affective regions and regions processing cognition and salient stimuli. Relative to pre-CBT in depressed adolescents, post-CBT functional connectivity significantly increased between the amygdala and the left dorsolateral PFC, bilateral dorsal ACC, and the left anterior insula. Changes in connectivity correlated with significant pre-to-post CBT symptom improvement, and pre-treatment amygdala connectivity predicted treatment response in depressed adolescents
Traynor et al. (2018)	62	10–21	Autism	Resting state fMRI	Diagnosis	Group difference	Compared to controls, ASD adolescents showed negative connectivity of the PCC with the angular gyrus, positive connectivity of the PCC with the superior temporal gyrus, over-connectivity of the hippocampus with the associative visual cortex, over-connectivity of the thalamus with multiple sensory processing areas of the cortex, over-connectivity of basal ganglia structures (putamen and globus pallidus) with somatosensory and motor cortices and with the fusiform gyrus, and under-connectivity of the left hippocampus with the right peri-rhinal cortex. Within the ASD group, a significant positive association was found between total RBS-R (Repetitive Behavior Score—Revised) score and connectivity between the left primary visual cortex and right inferior frontal gyrus, pars orbitalis
Tymofiyeva et al. (2019)	30	13.2–17.8	Depression	DTI and tractography	Predicting treatment response	Single subject	Machine learning classification applied to DTI-based structural connectome resulted in an 83% accuracy of predicting depressive symptom reduction with CBT
Tymofiyeva et al. (2017)	98	13–17	Depression	DTI and tractography	Diagnosis	Group difference	Compared to healthy controls, depressed subjects showed significantly lower FA-weighted node strength of the right caudate. FA-weighted node strength was correlated positively with age across both groups. Network-Based Statistic analysis showed a cluster of lower FA-based connectivity in depressed subjects centered on the right caudate, including connections to the frontal gyri, insula, and anterior cingulate. Within this cluster, the strongest difference between controls and depressed subjects was the connection between the right caudate and middle frontal gyrus, which showed a significant diagnosis by stress interaction and a negative correlation with total stress in depressed subjects
Velasquez et al. (2017)	108	8–19	Autism	Task-based fMRI (face processing task)	Diagnosis	Group difference	During face processing tasks, ASD youth with low-expressing 5-HTTLPR (serotonin transporter-linked polymorphic region) genotypes showed significantly greater amygdala-sgACC connectivity compared to healthy controls and to ASD youth with higher-expressing genotypes. ASD youth with higher-expressing genotypes also showed a negative relationship between amygdala-sgACC connectivity and social dysfunction
Wang et al. (2018)	136	21.5 ± 4.2 (control) 21.7 ± 3.6 (ARMS-NT) 19.7 ± 3.1 (ARMS-T)	Psychosis	Resting state fMRI	Future risk	Group difference	Subjects with At Risk Mental State (ARMS) that transitioned to psychosis (ARMS-T) during follow-up showed significant reductions in functional connectivity at baseline, primarily involving the limbic system, in comparison to healthy controls and to ARMS subjects that did not transition (ARMS-NT). ARMS-T subjects also exhibited reduced global efficiency at baseline in comparison to ARMS-NT subjects. In the salience network, the mean nodal efficiency computed over regions that were reduced in the ARMS-T group was associated with baseline positive and negative syndrome scale (PANSS) general scores. At the whole brain level, the ARMS-T group network community structure displayed a distinct pattern from that of the ARMS-NT group and of healthy controls
Wang et al. (2018a)	79	13–18	Schizophrenia	Resting state fMRI	Diagnosis	Group difference	Compared to healthy controls, patients with adolescent-onset schizophrenia had increased long-range and short-range positive fc in the right middle frontal gyrus and right superior medial PFC within the anterior DMN, decreased long-range and short-range positive fc in several regions of the posterior DMN, and decreased long-range positive functional connectivity within the SN. Decreased long-range positive fc in the left superior temporal gyrus was positively correlated with cognitive impairment
Wang et al. (2018b)	79	13–18	Schizophrenia	Resting state fMRI	Diagnosis	Single subject Group difference	Patients with adolescent-onset schizophrenia showed significantly increased regional homogeneity (local fc) values in the bilateral superior medial PFC and significantly decreased values in the left superior temporal gyrus, right precentral lobule, right inferior parietal lobule (IPL), and left paracentral lobule when compared to controls. A combination of the regional homogeneity values in the bilateral superior medial PFC, left superior temporal gyrus, and right IPL was used to discriminate patients from healthy controls
Wang et al. (2017)	79	13–18	Schizophrenia	Resting state fMRI	Diagnosis	Group difference	Compared to healthy controls, patients with adolescent-onset schizophrenia showed significantly increased functional connectivity strength in the left cerebellum VI and right inferior frontal gyrus/insula. Functional connectivity strength values in the right inferior frontal gyrus/insula positively correlated with general psychopathology scores of positive and negative syndrome scale
Wolf and Herringa (2016)	48	8–18	PTSD	Task-based fMRI (facial emotion processing task)	Diagnosis	Group difference	Among youth with PTSD, dorsomedial PFC activation and amygdala-medial PFC connectivity were inversely related to PTSD severity
Yao et al. (2017)	52	16.85 ± 0.60 (A) 16.56 ± 0.96 (control)	Anxiety	Resting state fMRI	Diagnosis	Single subject Group difference	This study used temporal features derived from dynamic fc to identify generalized anxiety disorder in adolescents. Instantaneous synchronization of pairwise signals was estimated as dynamic fc, and the Hurst exponent (regularity of a time series) and variance (variable degree of a time series) were calculated as temporal features of dynamic fc. By leave-one-out cross-validation (LOOCV), an accuracy of 88.46% was achieved when Hurst exponent and variance of dynamic fc were combined as features. Disease-related regions were also identified, including regions belonging to the DMN and cerebellar network
You et al. (2020)	31	9–13	Autism	Resting state fMRI Task-based fMRI (sustained attention task)	Diagnosis	Group difference	Distant fc of regions in the left frontal lobe, right parietal lobe, and left posterior middle temporal cortex showed a group by state interaction such that in the task state (relative to the resting state), fc was reduced (became focal) in healthy controls but increased (became diffuse) in children with ASD. In ASD children, higher state-related increase in distant connectivity of the left frontal and right angular gyrus predicted worse inattention, and global efficiency and modularity were also sensitive to group by state differences, with the magnitude of state-related change predicting inattention
Zhang et al. (2016)	100	15–25	Depression (suicidality)	Resting state fMRI	Diagnosis Future risk	Group difference	Compared to healthy controls, depressed patients showed increased fc in select DMN regions. Among depressed patients, suicidal patients showed increased connectivity in the left cerebellum and decreased connectivity in the right PCC, whereas non-suicidal patients showed increased connectivity in the left superior frontal gyrus, left lingual gyrus, and right precuneus, and decreased connectivity in the left cerebellum. No differences in the scores of any clinical scales were found between suicidal and non-suicidal depressed patients

*C, controls; ADHD, attention-deficit/hyperactivity disorder; ASD, autism spectrum disorder; OCD, obsessive-compulsive disorder; PTSD, post-traumatic stress disorder; CBT, cognitive-behavioral therapy; fMRI, functional MRI; fc, functional connectivity; rsFC, resting state functional connectivity; DTI, diffusion tensor imaging; FA, fractional anisotropy; ACC, anterior cingulate cortex; sgACC, subgenual anterior cingulate cortex; PFC, prefrontal cortex; OFC, orbitofrontal cortex; PCC, posterior cingulate cortex; DMN, default mode network; CEN, central executive network; SN, salience network*.

Of the 96 included articles (Table 1), 77 studied clinical populations (MDD, autism, anxiety, bipolar disorder, schizophrenia, ADHD, PTSD, OCD), whereas 19 assessed psychiatrically relevant constructs in non-clinical populations. For example, multiple studies demonstrated disruption in structural and functional connectivity in adolescents with MDD compared to controls in fronto-striatal, fronto-limbic, anterior cingulate cortical (ACC), insular, and amygdalar networks (Ho et al., 2014; LeWinn et al., 2014, 2018; Pannekoek et al., 2014; Davey et al., 2015; Henje Blom et al., 2015; Kim et al., 2016; Morgan et al., 2016; Chattopadhyay et al., 2017; Ellis et al., 2017; Straub et al., 2017; Tymofiyeva et al., 2017). Four studies identified circuitry predictive of treatment response in depressed teens (Jacobs et al., 2016; Straub et al., 2017; Klimes-Dougan et al., 2018; Tymofiyeva et al., 2019). These data suggest that MR imaging biomarkers based on connectivity between key brain regions may offer guidance for treatment selection for depressed adolescents.

Of the 96 included articles, 19 investigated constructs associated with mental illness such as increased rumination, decreased resilience, sensitivity to loss, increased MDD symptom expression, social anxiety, decreased mindfulness, hyperactivity, inattention, anhedonia, etc., without explicitly studying DSM-diagnoses in adolescents. We find the contribution of these studies relevant to the topic because of the limitation of the current diagnostic system and potential of alternative approaches such as the National Institute of Mental Health (NIMH) Research Domain Criteria (RDoC), as will be discussed in the following section. Arguably the most important implication of these neuroimaging findings is that they provide both the motivation and the rationale to pursue a much more explicitly preventative psychiatry approach in helping at-risk children before they present with a manifest psychiatric disorder (McCrory et al., 2017). Developing a neurocognitively informed screening tool capable of accurately indexing latent vulnerability is essential if we are to identify those children who are not yet overtly symptomatic but who are at most risk for the development of a future psychiatric disorder. More broadly, by establishing a better understanding of the specific neurocognitive mechanisms implicated in the pathogenesis of psychiatric disorders, we will be in a much better position to develop effective preventative interventions that increase the likelihood of resilient outcomes in children and adolescents (McCrory et al., 2017).

**Figure 1:**
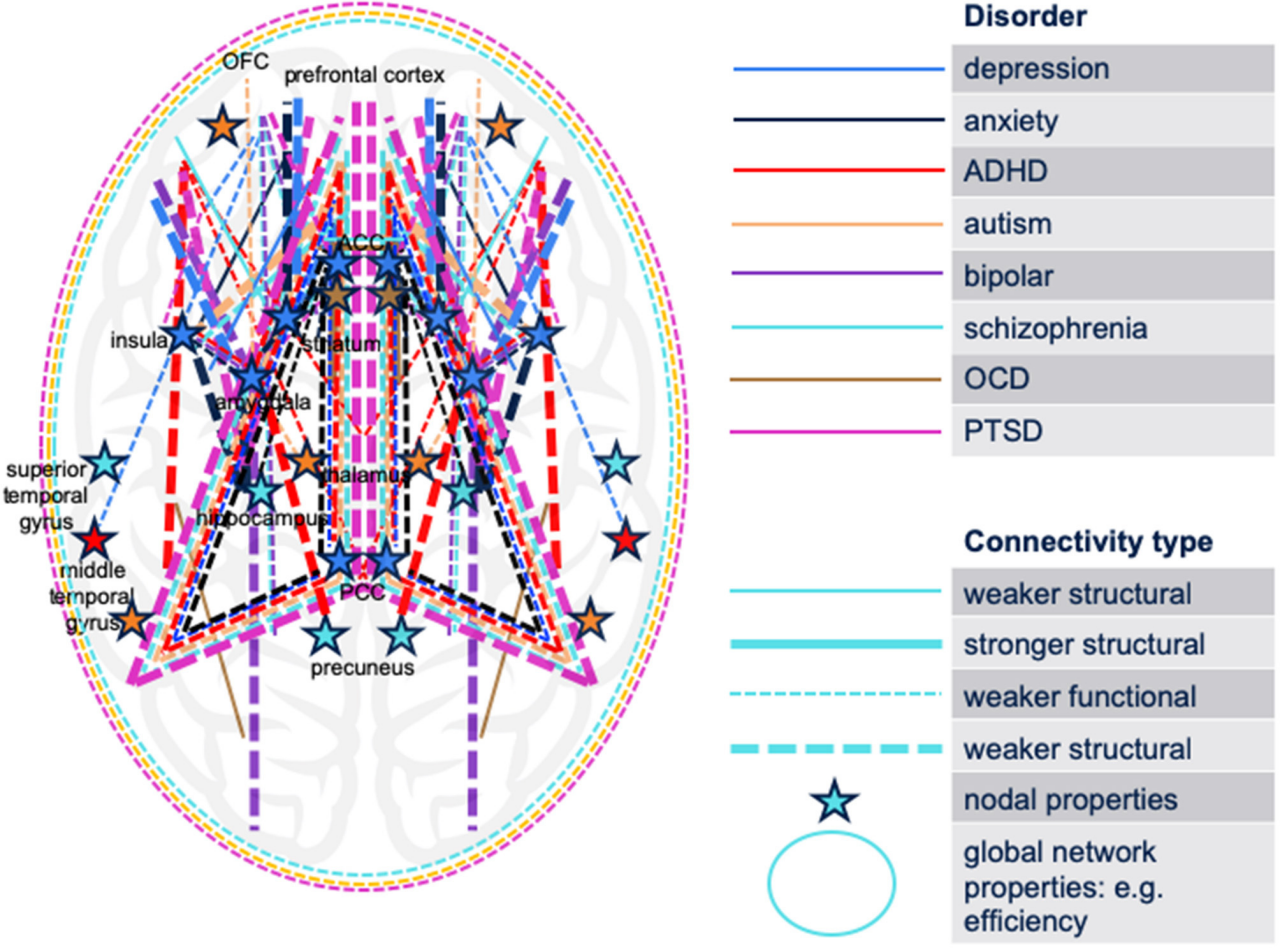
Schematic synthesis of the findings of the reviewed literature: a map of the brain circuitry implicated in eight psychiatric disorders (MDD, autism, anxiety, bipolar disorder, schizophrenia, ADHD, PTSD, OCD) or their relevant non-clinical symptoms in youth. Both increased and decreased structural and functional connectivities differentiate symptomatic subjects from controls. The cerebellum is not displayed. No differentiation between hemispheres is displayed. ADHD, attention-deficit/hyperactivity disorder; OCD, obsessive-compulsive disorder; PTSD, post-traumatic stress disorder; ACC, anterior cingulate cortex; OFC, orbitofrontal cortex; PCC, posterior cingulate cortex.

To graphically synthesize the findings of the reviewed literature, we have mapped the circuitry implicated in eight psychiatric disorders (MDD, autism, anxiety, bipolar disorder, schizophrenia, ADHD, PTSD, OCD) or their relevant non-clinical symptoms in youth onto a single brain model (Figure 1). Figure 1 illustrates both increased and decreased structural and functional connectivities as well as network properties that differentiate symptomatic subjects from controls. Similar to the model proposed by Williams (2017), connectivity aberrations associated with the disorders can be broadly classified as aberrations in six large-scale brain networks: default mode network (DMN), salience network (SN), threat network, reward network, attention network, and cognitive control network (CCN). Notably, this was observed not only for the mood disorders as proposed by Williams but also for other disorders, such as schizophrenia and autism (specifically associated with aberrations in DMN and SN) (Chen et al., 2017; Joshi et al., 2017; Wang et al., 2018a).

## Limitations of the Included Studies

### Small Sample Sizes

The evidence from the reviewed literature requires further investigation of the predictive and diagnostic potential of MRI connectivity measurements as the number of studies and sample sizes are very small. For example, seven of the reviewed studies included under 30 subjects per group. While small studies may be instrumental in finding new candidate biomarkers, replication of the results is the key. Additional well-planned imaging studies with large sample sizes are required. Yet, the field is developing rapidly. Large-scale collaborative efforts by consortia such as ABCD (Casey et al., 2018), ENIGMA (Thompson et al., 2014, 2017), and IMAGEN (O'Halloran et al., 2018) will help identify robust brain connectivity signatures of psychopathologies in adolescents. Among the included study, one study (Kaczkurkin et al., 2018) reports functional connectivity analyses using resting-state functional MRI in 833 participants who received both arterial spin labeling (ASL) and resting-state imaging as part of the Philadelphia Neurodevelopmental Cohort. The results revealed that overall psychopathology was associated with decreased connectivity between the dorsal ACC and bilateral caudate.

### Group Differences vs. Single-Subject Prediction

Another limitation of the psychiatric imaging literature in general is a profusion of statistically significant, but minimally differentiating biological findings (Kapur et al., 2012). In other words, thousands of studies are published on different aspects of brain disorders to show aberrations of some features (structural or functional) in a patient group usually in comparison with a healthy cohort. While these studies are valuable in terms of finding relevant disease biomarkers, they are not sufficient for direct clinical diagnostic/predictive adoption (Arbabshirani et al., 2017). The main reason is that many of these findings are statistically significant at the group level, but the individual discrimination ability of the proposed biomarkers is not typically evaluated. Group analysis aims to estimate the probability of a certain biomarker given the group (e.g., healthy controls or a patient group): *P[brain biomarker|group]*, and it is typically performed using general linear modeling (e.g., *t*-test or ANOVA). Single-subject prediction, on the other hand, predicts belonging to the group given the brain biomarker: *P[group|brain biomarker]*, and it is typically performed using generalized linear model such as logistic regression or artificial intelligence (AI) approaches such as machine learning. As mentioned previously, only group analysis is commonly performed in neuroimaging studies. In such cases, effect size can serve as an important indicator of the individual discrimination ability of the biomarker, but it is often not reported—as it is with the majority of the studies reviewed in Table 1. For continuous data analysis, the correlation coefficient points at high or rather low discrimination ability, as in the example of the large study by Kaczkurkin and colleagues discussed above (Kaczkurkin et al., 2018), where Pearson *r* = −0.18 and *r* = −0.15. The effect size is low if the value of r varies around 0.1, medium if r varies around 0.3, and large if r varies more than 0.5 (Rosenthal and Rosnow, 1984; Cohen, 1988). Since classification provides information for each individual subject, it is considered a much harder task than reporting group differences. Nevertheless, recent extensive evidence shows the great potential of neuroimaging data for single subject prediction of various brain disorders in adults (Arbabshirani et al., 2017). Several of the studies in adolescents also performed single-subject prediction analysis. For example, functional brain connectivity developmental patterns were found to be a reliable biomarker of severe attention impairment in youth, with a peak receiver operating characteristic curve of 79.3%, measured by area under the curve (Kessler et al., 2016). In another example, machine learning was applied to structural connectivity data to predict symptom improvement in depressed adolescents in response to cognitive behavioral therapy (CBT), resulting in an accuracy of 83% (Tymofiyeva et al., 2019).

### Use of the DSM Classification as Ground Truth

One fundamental challenge is that the reviewed clinical studies used the DSM-based diagnosis as the ground truth. The reliance on the categorical system of diagnosis has clear utility; however, its validity has been questioned and dimensional views of illness that incorporate continua of neurobiology and observable behavior have been proposed—i.e., NIMH RDoC (Insel et al., 2010). If one takes seriously the possibility of 1 day developing a neurobiologically-based diagnostic system that would replace the symptom-based DSM nosology, then one biases the results by using DSM diagnosis as the ground truth in research studies. Thus, the advancement of psychiatry appears to be hindered by circuitous reasoning (i.e., a “Catch-22”), and this circularity impedes the development of a clinically viable alternative system (Kapur et al., 2012). Importantly, a recent paper by Drysdale et al. reported promising results showing that adult depression can be subdivided into biological types (Drysdale et al., 2017). Specifically, functional MRI scans of more than 1,100 patients with clinical depression and healthy individuals enabled researchers to demonstrate that patients with depression can be divided into four subtypes based on distinct patterns of functional connectivity in limbic and fronto-striatal networks and different clinical symptoms (Drysdale et al., 2017). Notably, these four subtypes of depression were also associated with differences in clinical treatment outcome. While the study by Drysdale and colleagues demonstrated subtypes within one DSM category (adult MDD; Drysdale et al., 2017) neurobiologically-based diagnostic categories may easily span outside of the established DSM categories. Unsupervised clustering analyses in large datasets of community samples may help solve this problem and establish a classification system of connectome-based psychiatric disorders. In a study by Van Dam et al., data-driven approaches for identifying homogenous subgroups, spanning typical function to dysfunction, not only yielded clinically meaningful groups, but also captured behavioral and neurobiological variation among healthy individuals (Van Dam et al., 2017).

## Vision: the Role of Neuroimaging in Pediatrics

Here, we would like to present a vision of the role that neuroimaging may play in pediatrics and primary care in the future. We would like to start by presenting a case example of a clinical presentation of an adolescent patient to a primary care outpatient clinic.

*A concerned parent brings to the primary care clinic a 15-year-old adolescent male with a recent history of increased mood lability, irritability and changes in his personality that has led to significant difficulties with the patient's relationship with his parents and siblings at home and the patient's teachers and classmates at school. The parent states that the patient's personal hygiene has declined at home and that he has started to withdraw from his relationships with his family and friends. The parent also reports that the patient at times loses his temper and has been oppositional with his family, teachers and friends. During the interview by the primary care provider, the adolescent patient complains of problems with feeling easily irritated by others, difficulties with sleep and concentration, and headaches. There are times that the patient also reports feeling “on top of the world” and that he can accomplish many great things. The parent informs the primary care provider that there is a history of mood disorders in the family including a parent with depression and a relative with possible bipolar disorder. The parent also states that there is a relative who has had significant problems with truancy and breaking rules whose behavioral problems started during adolescence. Finally, the parent reports that a great grandparent was hospitalized with a diagnosis of schizophrenia. The parent is concerned about the patient and has brought him to the primary care clinic for help with the son that the parent loves and cares for very deeply*.

At present, psychiatric diagnoses are based on symptoms and classified as per the DSM criteria. One significant complicating factor is that most of the DSM diagnoses have been developed for adults, but these same DSM criteria are being applied to make diagnoses in children and adolescents. Based on the presenting symptoms and DSM criteria, the adolescent patient's differential diagnosis is broad. The patient's presenting symptoms of mood lability, irritability, sleep, and concentration problems, and relationship difficulties with family, friends and teachers could be due to a diagnosis of depression or bipolar disorder. The patient's loss of temper and oppositional behavior could be due to a mood disorder such as depression or oppositional defiant disorder that may be the beginning of a possible conduct disorder. The patient's report of feeling at times “on top of the world” and that he can accomplish many great things could be reflective of normative adolescent development or due to bipolar disorder. The patient's decrease in personal hygiene and withdrawal from his family and friends may be due to a mood disorder such as depression or the beginning of another possible psychiatric disorder such as schizoaffective disorder or schizophrenia. Finally, the patient's changes in personality such as his mood lability and complaints of headaches may be due to a mood disorder such as depression or a brain mass such as a tumor. Each of these possible diagnoses has a different clinical prognosis and treatment. Proper diagnosis is essential to ensure that the optimal clinical treatment is selected and to prevent the further worsening of the patient's condition due to either the incorrect diagnosis and treatment or the potential side effects of the use of the inappropriate treatment due to the incorrect diagnosis. For example, if the patient's symptoms lead the clinician to incorrectly make a diagnosis of bipolar disorder based on DSM criteria, then the patient will often be started on a mood stabilizer (e.g., lithium) or atypical antipsychotic medication (e.g., olanzapine) that have several potentially negative side effects including significant weight gain and increased risk for the development of type 2 diabetes. In addition to the potential negative consequences of an incorrect diagnosis to the patient, an incorrect diagnosis can also lead to great costs to the patient, patient's family, and society. For example, missing the diagnosis of depression can lead to significant present and future consequences since adolescent depression confers a strong risk for adult major depressive disorder, increased cardiovascular risk, medical illnesses, disability, premature death, academic and work problems, relationship problems with family and friends, substance abuse, and suicide (Rao et al., 1993, 1995, 1999; Lewinsohn et al., 1998; Pine et al., 1998; Armstrong and Costello, 2002; Lehrer et al., 2006; Copeland et al., 2007; Crum et al., 2008; Audrain-McGovern et al., 2009; Rao and Chen, 2009; Maughan et al., 2013; Liu et al., 2017). Depression is a highly prevalent, devastating, costly and frequently re-occurring chronic illness that the World Health Organization (WHO) ranks as the #1 leading cause of disability worldwide, affecting over 300 million people (WHO, 2017) at an estimated cost of over $210.5 billion per year in the U.S. alone (Greenberg et al., 2015).

**Figure 2:**
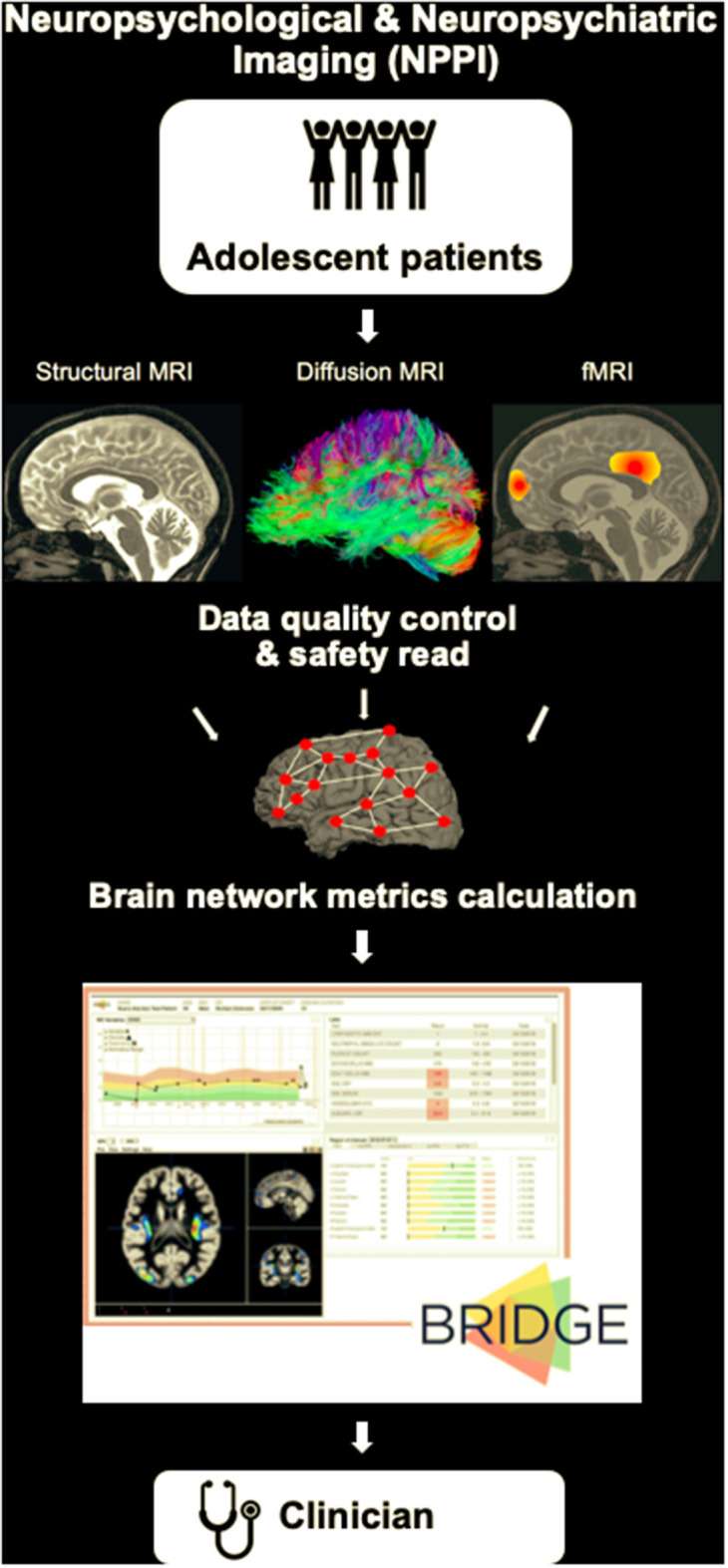
A schematic of our vision for a routine neuropsychological and neuropsychiatric imaging (NPPI) protocol for adolescent patients. The standardized MRI protocol could consist, for example, of a 30 min brain scan at a 3T MRI scanner that would include a localizer scan, a T1-weighted sequence, a 55-direction diffusion-weighted sequence and an eyes-closed resting-state fMRI sequence. The MRI scan will be followed by a quality control and safety read of the scan by a radiologist. Next, connectivity matrices will be derived, and structural and functional brain network metrics will be calculated. Platforms like BRIDGE (https://bridge.ucsf.edu) can be used to integrate the obtained data and provide the output to the clinician (pediatrician) by means of organized dashboards.

Our vision for preventing many of the drastic consequences described in the case example is the introduction of a routine *neuropsychological and neuropsychiatric imaging (NPPI)* protocol for adolescent patients (Figure 2). The rationale for the presented vision is rooted in the research successes described above, with the expectation that the outlined limitations can be overcome with dedicated work of researchers around the world. The standardized protocol could consist of the following:

- A 30 min brain scan at a 3T MRI scanner that would include a localizer scan, a T1-weighted sequence, a 55-direction diffusion-weighted sequence and an eyes-closed resting-state fMRI sequence.
- A quality control and safety read of the scan by a radiologist.
- Network construction and a computer-based calculation of the structural and functional brain network metrics (e.g., using the Brain Connectivity Toolbox brain-connectivity-toolbox.net) (Rubinov and Sporns, 2010) that can be compared to the normative data by the pediatrician (e.g., using a platform like BRIDGE https://bridge.ucsf.edu).

The chosen MRI sequences are an example of an easily standardizable set of sequences that in a short time could provide structural and functional connectivity data of sufficient quality. The choice of the 55-direction diffusion-weighted sequence is based on its suitability for high angular resolution diffusion imaging (HARDI) analyses that can help resolve crossing fibers (Tuch et al., 2002). The resting-state fMRI sequence can realistically be performed at any imaging center, and it can potentially allow for derivation of task-based information (Tavor et al., 2016).

The protocol can be offered to all adolescent patients without MRI contraindications (e.g., cardiac pacemakers, braces, etc.) to meet two goals:

- To help generate the differential diagnosis for an individual patient or assess risk.
- To personalize treatment (predict treatment outcome) for an individual patient.

For improved diagnostic/predictive accuracy, the scan can be potentially conducted at two different times or even once per year during the adolescent years: 14y.o., 15y.o., 16y.o., 17y.o. (Kessler et al., 2016), especially since the age of onset of different psychiatric disorders can vary. Normative modeling can provide a way to map deviations from an expected pattern at the individual level (Kessler et al., 2016; Marquand et al., 2019). Whereas identifying MRI-based “biotypes” as an alternative to DSM categories is one approach to make diagnosis using NPPI in the future, another approach would be to measure network aberration along several continuous NIMH RDoC dimensions and creating profiles that would inform risks similar to high blood pressure in cardiology, and inform clinical care by suggesting the optimal treatment choice for an individual patient (Williams, 2017).

While there are multiple steps necessary to implement this vision, the common concern that is raised when considering imaging is the cost of the MRI procedure, which we will address in the following section.

## Cost-Benefit Analysis: MDD as an Example

Here, we present a cost-benefit analysis of conducting routine NPPI in adolescents using only a single DSM diagnosis, MDD, as an example (Supplementary Figure 1).

Adolescent depression can be a significant economic burden to patients, families, and society. A cost-of-illness study on adolescent depression in the U.S. estimates direct costs of $1,120 and indirect costs of $310 per year, when accounting for the effects of school absences and parental lost days at work (Domino et al., 2009; Beecham, 2014). Furthermore, adolescents with depression are likely to have higher healthcare costs in other domains, due to more contacts with other healthcare providers (Lynch and Clarke, 2006). These ramifications often continue into adulthood as individuals who had depression in adolescence continue to be associated with higher healthcare utilization and increased work impairment in young adulthood (Keenan-Miller et al., 2007). Costs of adult depression in the U.S. are over $210.5 billion annually (Greenberg et al., 2015). This suggests an economic argument for timely identification and treatment of adolescent depression and for considering the costs of adult depression when weighing costs of MR imaging against benefits.

We estimate the costs of a NPPI scan at $400 per patient. This estimate is based on the University of California San Francisco (UCSF) 3T MRI external recharge rates: $350/30 min (https://radiology.ucsf.edu/research/core-services/7T-3T-MB) + $50–75 for reading for incidental findings (*estimate provided by Research Radiology, San Francisco, USA*). While scheduling short 30 min time slots at an MRI scanner may currently not be possible everywhere, streamlined procedures and even cost-reducing solutions such as dedicated head-only scanners (Foo et al., 2018) can be leveraged to increase throughput in the future. In contrast to a conventional MRI, the scan will not need to be read diagnostically by a neuroradiologist; instead a neuroradiologist will need to perform image quality control and a “safety read” for incidental findings. The brain network metrics will be calculated using a computer algorithm. In case of incidental findings, such as an indication of a tumor, further examination and/or treatment may be indicated. The effectiveness of the NPPI will depend on the sensitivity and specificity of the biomarkers. An accuracy of 80% (both sensitivity and specificity) is generally considered to characterize a clinically useful biomarker (Savitz et al., 2013). The adolescent brain connectivity papers reviewed above where single-subject prediction analysis was performed displayed biomarker accuracy in this range (80–83%) (Kessler et al., 2016; Tymofiyeva et al., 2019). Importantly, several single-subject prediction studies in adults that demonstrated MRI's potential to predict treatment response also studied for comparison the predictive value of demographic and clinical variables—as these variables are more readily available and would be a cheaper solution (Månsson et al., 2015; Thompson et al., 2015; Drysdale et al., 2017). These studies found that these demographic and clinical variables *failed* as predictors of clinical improvement after treatment (Månsson et al., 2015; Thompson et al., 2015; Drysdale et al., 2017).

Based on U.S. Census Bureau estimates, there were 4.1 million 14-year-olds in the U.S. in 2017 (US Census Bureau, 2017). If we performed a routine NPPI scan on every 14-year-old in the U.S. annually, we estimate a cost of $1.6 billion per year, based on an estimated cost of $400 per MRI scan. The estimated cost of adult MDD in the U.S. is over $210.5 billion annually (Greenberg et al., 2015). This means that NPPI would only have to help prevent (through early detection and personalized treatment) ~0.8% of adult MDD cases to completely cover its costs. Adolescence is the optimal time to intervene because it is when MDD often begins, and it increases the risk of adult depression by a factor of 2- to 3-fold (Pine et al., 1998).

In the calculation above we assumed that we would routinely scan *every* 14-year-old. This is an extreme scenario, whereas a more plausible scenario would be to scan only at-risk youth and youth presenting with clinically significant problems. This more targeted approach would reduce the estimated total cost of NPPI that we presented above. Given the significant costs of teen MDD to the patient, family and society, the cost of an MRI scan is well worth the benefit in terms of decreased prolonged emotional pain and suffering, lower risk of adult MDD and healthcare costs, and improved health and productivity of the patient. As discussed above, among many other problems, MDD elevates the risk for cardiovascular disease in both adolescents (Goldstein et al., 2015) and adults (Penninx, 2017), and MDD increases the medical costs of treating and managing primary care illnesses such as diabetes in adolescents (Stewart et al., 2005). Additionally, MRI scans are routinely done for medical conditions (e.g., lower back pain and knee injuries) that do not have the potentially devastating consequences of MDD (e.g., suicide). Another example is routine prenatal ultrasound imaging visits that are done with all, not just at-risk, pregnant women. Thus, once valid and reliable MRI biomarkers have been developed and tested for teen MDD, the cost of an MRI scan should not be a justifiable impediment since MRI scans are frequently performed for much less costly and devastating medical conditions. Medical insurance groups or Health Maintenance Organizations (HMOs) may consider this approach as a means of reducing overall healthcare costs, since early prevention could reduce the incidence of adult MDD, which is very costly to both the patient and healthcare system. We also wish to emphasize that our conservative estimate only focuses on MDD; however, many other psychiatric conditions can be potentially assessed using the same MRI scan.

## The Roadmap

What is needed for this vision to be implemented? We briefly present below a roadmap of the steps required for this vision to be implemented.

- *A large normative database*. As discussed above, the field is moving toward collecting big data (that often include a replication sample). The ABCD study has already started to release MRI neuroimaging datasets for 10,000 youth who will be followed longitudinally and scanned every 2-years over a total duration of 10-years. As mentioned above, other initiatives such as ENIGMA and IMAGEN can also provide important big datasets.
- *A set of NPPI-derived metrics, their sensitivity/specificity, and guidelines on how to combine them with symptom-based information*.
- *Consensus guidelines for when NPPI is indicated* (expert consensus panel).
- *Required qualifications and pipeline* for *NPPI* + *data quality and safety read* + *metrics calculation*. There is currently a high heterogeneity of analysis pipelines used by researchers to derive brain connectivity matrices, which can lead to large discrepancies between the resulting structural (Qi et al., 2015) and functional (Carp, 2012) network metrics. In addition, choice of brain parcellation and edge weights will also affect test-retest reliability of the resulting metrics (Cammoun et al., 2012; Yuan et al., 2019). Development of a standardized, objective, and publicly available pipeline is a necessary step. Once the connectivity matrices are derived, network metrics can be calculated, e.g., using the Brain Connectivity Toolbox (brain-connectivity-toolbox.net) (Rubinov and Sporns, 2010). The Brain Connectivity Toolbox is a MATLAB toolbox for complex-network analysis of structural and functional brain connectivity datasets, which is widely used by brain-imaging researchers and has been incorporated in many projects, including the Human Connectome Project. Platforms like BRIDGE (https://bridge.ucsf.edu) can be used to integrate the obtained data and provide the output to the clinician by means of organized dashboards. Required qualifications for data quality assessment and safety reads need to be specified.
- *CPT code*. The Current Procedural Terminology (CPT) code set is a medical code set maintained by the American Medical Association through the CPT Editorial Panel. This step is required to enable insurance companies to pay for adolescent NPPI (e.g., similar to ultrasound for neonates).

## Discussion

At present, utilization of neuroimaging biomarkers for clinical practice is restricted to neurological conditions such as pre-surgical evaluation of epilepsy, differential diagnosis of coma, and brain-computer interfaces for locked-in patients (Arslan, 2018). For psychiatric conditions, this is yet to be established for routine clinical applications. Because adolescence is an especially vulnerable time for the development of many important psychiatric disorders, this period presents an especially important opportunity to clinically intervene. In this perspective article, we assess the MRI-based brain connectivity literature over the last 5-years that provides insights into development of psychiatric disorders in adolescents. While the subject numbers are still small and the focus on group differences as opposed to individual subject-based predictions prevails, the reviewed literature demonstrates the potential of MRI to diagnose psychiatric disorders, predict their development, and predict response to clinical treatment. We believe that the continuous progress in neuroimaging techniques together with the ongoing well-coordinated large-scale neuroimaging studies of adolescent brain development will help identify robust biomarkers for personalized/stratified medicine (Kapur et al., 2012) with a key focus on prevention. Inspired by this significant potential, we offer a vision for the role that neuroimaging may play in pediatrics and primary care in the future: a routine NPPI protocol for adolescent patients that could save significant costs to the patients, their families, and society, and significantly reduce suffering that often results from undiagnosed and misdiagnosed adolescent psychiatric disorders. The proposed vision can also help offer preventative measures to at-risk youth in a targeted manner—e.g., by recommending the Training for Awareness, Resilience and Action (TARA) (Henje Blom et al., 2014, 2017) to youth at risk for developing depression. Insurance companies are more likely to reimburse such preventative measures when numerical cut-offs are used, as will be provided by our proposed NPPI. The roadmap we provide in paper can help accomplish this endeavor.

## Data Availability Statement

The original contributions presented in the study are included in the article/Supplementary Material, further inquiries can be directed to the corresponding author(s).

## Author Contributions

OT, DX, CH, and TY conceptualized the article. VZ performed literature search. OT, C-ML, and TY performed cost-benefit analysis. OT created figures. OT, VZ, C-ML, DX, CH, and TY wrote the manuscript. All authors contributed to the article and approved the submitted version.

## Conflict of Interest

The authors declare that the research was conducted in the absence of any commercial or financial relationships that could be construed as a potential conflict of interest.
