# Supplementary figures and images for: MRI Insights Into Adolescent Neurocircuitry—A Vision for the Future
Source: Front Hum Neurosci. 2020 Jul 7;14:237. doi: 10.3389/fnhum.2020.00237 (PMC7359264; doi:10.3389/fnhum.2020.00237)

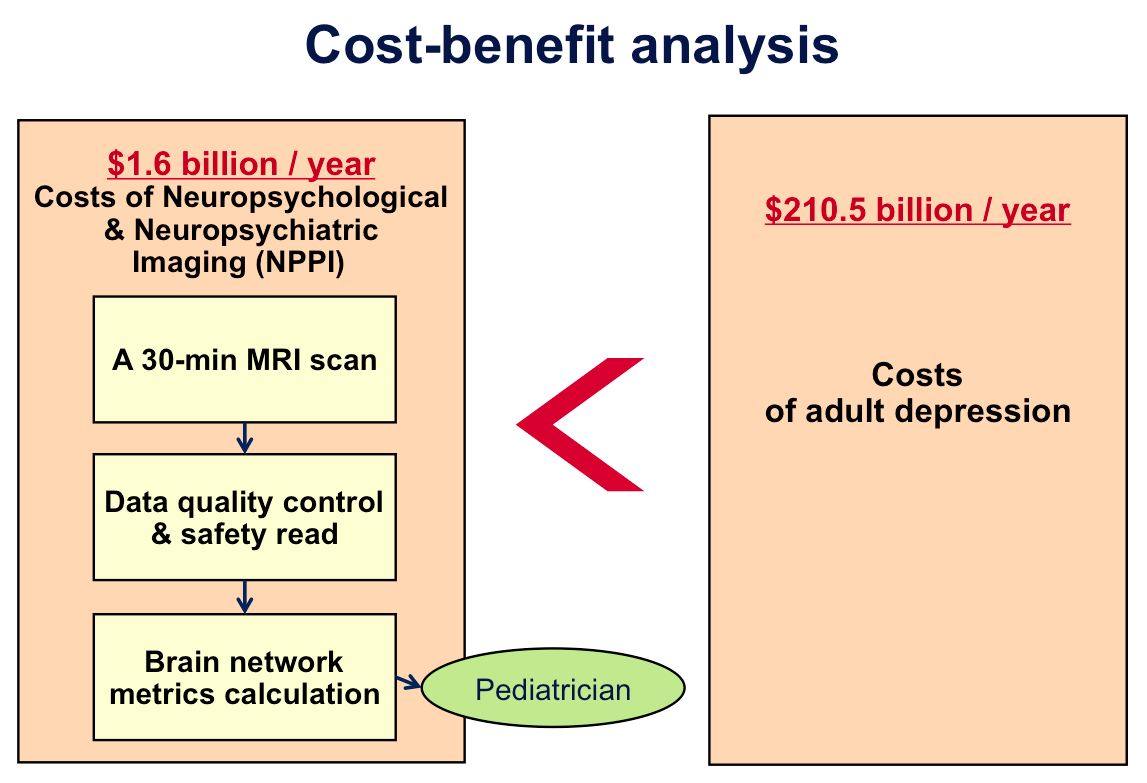

Supplement: Supplementary file 1 [file Image_1.TIFF]
